# Antipsychotic drug use complicates assessment of gene expression changes associated with schizophrenia

**DOI:** 10.1038/s41398-023-02392-8

**Published:** 2023-03-17

**Authors:** Anton Schulmann, Stefano Marenco, Marquis P. Vawter, Nirmala Akula, Agenor Limon, Ajeet Mandal, Pavan K. Auluck, Yash Patel, Barbara K. Lipska, Francis J. McMahon

**Affiliations:** 1 Human Genetics Branch, National Institute of Mental Health Intramural Research Program, Bethesda, MD USA; 2 Human Brain Collection Core, National Institute of Mental Health Intramural Research Program, Bethesda, MD USA; 3 Functional Genomics Laboratory, Department of Psychiatry & Human Behavior, University of California, Irvine, Irvine, CA USA; 4 Department of Neurology, University of Texas Medical Branch at Galveston, Galveston, TX USA

**Keywords:** Schizophrenia, Molecular neuroscience

## Abstract

Recent postmortem transcriptomic studies of schizophrenia (SCZ) have shown hundreds of differentially expressed genes. However, the extent to which these gene expression changes reflect antipsychotic drug (APD) exposure remains uncertain. We compared differential gene expression in the prefrontal cortex of SCZ patients who tested positive for APDs at the time of death with SCZ patients who did not. APD exposure was associated with numerous changes in the brain transcriptome, especially among SCZ patients on atypical APDs. Brain transcriptome data from macaques chronically treated with APDs showed that APDs affect the expression of many functionally relevant genes, some of which show expression changes in the same directions as those observed in SCZ. Co-expression modules enriched for synaptic function showed convergent patterns between SCZ and some of the APD effects, while those associated with inflammation and glucose metabolism exhibited predominantly divergent patterns between SCZ and APD effects. In contrast, major cell-type shifts inferred in SCZ were primarily unaffected by APD use. These results show that APDs may confound SCZ-associated gene expression changes in postmortem brain tissue. Disentangling these effects will help identify causal genes and improve our neurobiological understanding of SCZ.

## Introduction

Recent large-scale transcriptome studies have identified hundreds to thousands of differentially expressed genes in the dorsolateral prefrontal cortex (DLPFC) of individuals diagnosed with schizophrenia (SCZ) [1, 2]. Functional interpretation of these massive transcriptional changes remains an ongoing effort. Postmortem transcriptome data is often highly confounded, requiring extensive correction for various technical and demographic variables. Antipsychotic drug (APD) use is one potential confounder that is particularly difficult to account for. Since nearly all SCZ patients are exposed to APDs and severely ill patients are exposed to more APDs, the effects of APDs on postmortem human brain transcriptome are challenging to separate from those of SCZ itself. Despite recent attempts to assess the transcriptional impact of APDs on postmortem human brain [3, 4], the effects of APDs on the prefrontal cortex transcriptome and their relationship with SCZ remain poorly understood. Some evidence suggests that APDs induce gene expression changes consistent with those seen in SCZ [5–7]. In contrast, other studies indicate that APDs may drive the expression of some genes in opposite directions to those seen in SCZ [8–10]. Here we assessed the transcriptomic effects of APD use in SCZ using a novel approach that compared SCZ-associated gene expression changes between individuals taking APDs at the time of death with those who were not. We also examined transcriptome data from healthy macaques administered APDs and integrated these data with the human data. Our findings suggest that APD exposure contributes in complex ways to gene expression changes seen in SCZ, with important implications for the biological interpretation of findings from human postmortem brain studies.

## Results

### Atypical APD use is correlated with a more prominent SCZ signature

To investigate the effect of APD exposure on postmortem gene expression changes, we calculated an aggregate score of SCZ-associated differential gene expression—here referred to as the “SCZ expression signature” (see “Methods”)—across groups of DLPFC samples in the NIMH Human Brain Collection Core (HBCC) taken from donors diagnosed with SCZ. We grouped the samples by toxicology results detected in postmortem blood into samples that tested positive (*n* = 65) or negative (*n* = 23) for any APD, and further subdivided the APD-positive group into those where typical (*n* = 27), atypical (*n* = 28), or mixed (both types; *n* = 10) APDs were detected.

**Fig. 1:**
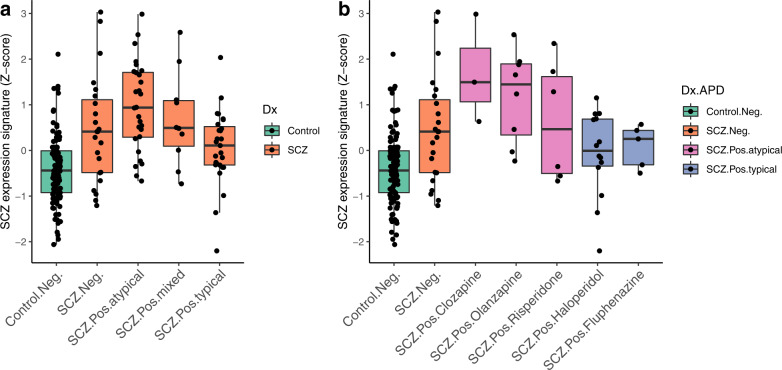
SCZ-associated differential gene expression between toxicological subgroups. **a** Aggregated scores of differential gene expression (SCZ expression signatures) are shown based on toxicological findings (Neg.: negative for any APD; Pos.: positive for atypical, typical, or both APD [mixed] classes). Kruskal–Wallis test (KWt) on all groups: *p* = 3.83e-10; KWt within SCZ subgroups only: *p* = 0.014. For details and post hoc Dunn tests, see Supplementary Table S2. **b** As in **a** but SCZ samples were divided into groups of 3 or more cases positive for a single APD (atypical APDs: clozapine [*n* = 3], olanzapine [*n* = 8], risperidone [*n* = 6]; typical APDs: haloperidol [*n* = 14], fluphenazine [*n* = 5]). SCZ expression signatures were based on gene expression values residualized for major influential covariates, such as sex, age, RNA integrity, and postmortem interval (see “Methods”).

APD exposure at the time of death was associated with numerous changes in the brain transcriptome. Individuals in the atypical APD group had the most pronounced differential gene expression changes compared to psychiatrically healthy controls, followed by the mixed APD, APD-negative, and typical APD groups (Fig. 1a). Further examination of those testing positive for a single APD compound revealed that samples with clozapine and olanzapine had the highest SCZ expression signature, followed by samples with risperidone, fluphenazine, and haloperidol (Fig. 1b). Similar results were obtained with the SCZ expression signature calculated based on the larger PsychENCODE dataset [2] (Supplementary Fig. S1) and in a smaller independent validation cohort with associated postmortem brain toxicology data from UC Irvine (Supplementary Fig. S2). Similarly, traditional differential gene expression analyses for SCZ subgroups showed that individuals with atypical APD exposure had the largest number of differentially expressed genes (Supplementary Table S1). These findings show that transcriptional differences between SCZ cases and controls are larger in APD-positive cases, particularly those treated with atypical APDs.

### SCZ expression signatures are correlated with APD signatures in macaques

Differential gene expression in the human brain can reflect both APD exposure and disease severity. To study the effects of APDs on gene expression in relative isolation, we used published transcriptome data from the DLPFC of healthy rhesus macaques treated with either clozapine, haloperidol, or placebo [11]. While the differential gene expression analysis did not yield any genes that passed a false discovery rate (FDR) threshold of 5%, a gene ontology analysis of the top nominally significant genes did show significant enrichment of terms related to immunological processes, metabolism, and synaptic transmission (Supplementary Fig. S3 and Supplementary Table S3). These functional enrichments are also similar to those previously found for haloperidol and clozapine in rodent forebrain tissue [12, 13]. A direct comparison between mouse and macaque APD effects showed high concordance for haloperidol and low but significant concordance for clozapine (Supplementary Fig. S4).

**Fig. 2:**
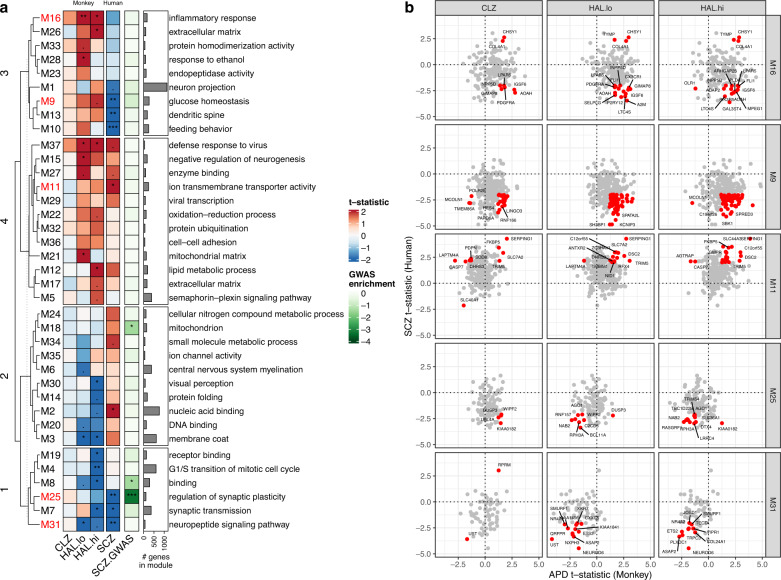
Consensus WGCNA between human and macaque DLPFC transcriptome data. **a** Relationship of module eigengenes to APD use in macaque (CLZ = clozapine, HAL.lo = low-dose haloperidol; HAL.hi = high-dose haloperidol) and humans with SCZ (data from HBCC). Heat map shows *t*-statistic from linear regression illustrating module eigengene directionality (same color indicates concordance; two-sided *t* test significance level. ***p < 0.001, **p < 0.01, *p < 0.05. Hierarchical clustering of rows with Euclidean distance cut at the level of four branches was used for better visibility. Heat map annotation shows the number of genes in each module and the most over-represented gene ontology term (based on Fisher’s exact test; excluding terms with ≤3 hits). SCZ.GWAS: Enrichment (Fisher’s exact test) of prioritized genes from the 2022 SCZ GWAS [15]. For details on the statistical tests and FDR-corrected *p* values, see Supplementary Table S2. **b** Relationships of individual genes to APD exposure in macaque (*x*-axis) and humans with SCZ (*y*-axis) for five example modules with predominantly divergent (M16, M9) or convergent (M11, M25, M31) gene expression patterns (shown as *t*-statistics). For visualization purposes, the top genes with nominal *p* < 0.05 for SCZ and *p* < 0.25 for each APD in macaques are highlighted in red. For a full list of genes and enriched gene ontology terms, see Supplementary Table S4.

To harness information across conserved, functionally relevant gene co-expression networks, we applied signed consensus WGCNA [14] across the macaque and human datasets. We identified 37 consensus modules. Relationships of these modules with APD exposure in macaques and SCZ in humans, along with functional enrichments, were examined (Fig. 2a). Some module eigengenes (e.g., M16, M9) were associated with APD exposure in the opposite direction from that observed in SCZ; this divergence is consistent with a “normalizing” effect of APDs in those modules. Other modules (e.g., M11, M25, M31) showed concordant association in the same direction for APD treatment and for SCZ, suggesting that treatment and disease are confounded in those modules (Fig. 2b). These convergent changes were primarily observed for macaques treated with haloperidol, while those treated with clozapine exhibited an inconsistent or divergent pattern for these modules. Notably, these modules were enriched for synapse-related terms, including a module (M25) enriched for genes prioritized in the most recent genome-wide association study (GWAS) of SCZ [15]. Modules enriched for inflammatory response (M16), glucose homeostasis (M9), and mitochondria (M18) exhibited a predominantly divergent pattern. In summary, APD exposure in monkeys induced changes in gene expression across distinct, functionally relevant, conserved gene networks that were both convergent and divergent with those seen in SCZ.

### Schizophrenia-related cell-type shifts are largely unaffected by APD exposure

**Fig. 3:**
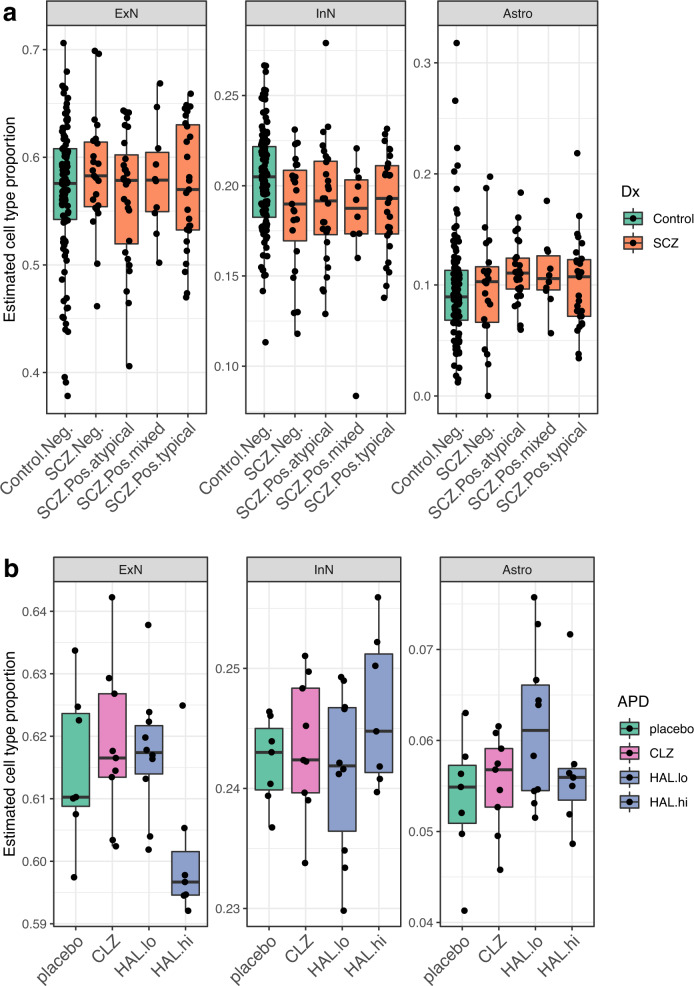
Estimated cell-type proportions for human and macaque DLPFC samples. **a** Cell-type proportions of excitatory neurons (ExN), inhibitory neurons (InN), and astrocytes (Astro) in bulk DLPFC tissue of SCZ cases with different toxicological profiles (groups as in Fig. 1a) estimated via corresponding single-nucleus RNA-seq profiles. Kruskal–Wallis test (KWt) on all groups: *p*_ExN_ = 0.908; *p*_InN_ = 0.0318; *p*_Astro_ = 0.0689; KWt within SCZ subgroups only: *p*_ExN_ = 0.798; *p*_InN_ = 0.875; *p*_Astro_ = 0.565. Mann–Whitney *U*-test (MWU) between SCZ and controls was significant for InN (*p* = 0.00159) and Astro (*p* = 0.0176). Only InN remained significant after FDR correction. **b** Estimated proportions of ExN, InN, and Astro in bulk DLPFC of monkeys treated with clozapine (CLZ), haloperidol (low dose: HAL.lo; high dose: HAL.hi), or placebo. KWt: *p*_ExN_ = 0.0433; *p*_InN_ = 0.547; *p*_Astro_ = 0.336. MWU between APDs and placebo was significant only for HAL.hi in ExN (*p* = 0.0379). For detailed test statistics, see Supplementary Table S2. For the other cell types, see Supplementary Fig. S5.

Prior studies have implicated cell-type shifts in SCZ pathogenesis [16, 17]. To examine if APD use may contribute to or reverse SCZ-associated cell-type shifts, we estimated cell-type proportions in the bulk transcriptome data based on single-nucleus reference data from the same brain bank (manuscript under review) using Bisque [18] and compared cases and controls based on their toxicological profile. This comparison revealed significant decreases in GABAergic neurons and nominally significant increases in astrocytes in the DLPFC of all SCZ samples. APD positivity at the time of death did not significantly affect cell-type proportions within the SCZ group (Fig. 3a and Supplementary Fig. S5c).

To study the effect of APD exposure alone on cell-type proportions, we also deconvolved DLPFC transcriptome data from macaques treated with APDs using single-cell data from adult macaque DLPFC [19]. We found no significant cell-type shifts except a slight decrease in glutamatergic neurons in animals treated with high-dose haloperidol, possibly indicating cytotoxic effects (Fig. 3b and Supplementary Fig. S5d). Together with the cell-type deconvolution results in humans, these data suggest that any inferred cell-type shifts observed in SCZ are likely unrelated to APD exposure.

## Discussion

We show that APD exposure at the time of death confounds SCZ-associated differential gene expression in postmortem brain. These findings suggest that some of the differential gene expression previously attributed to SCZ may in fact, be driven by APD exposure. This is especially true among individuals treated with the atypical APDs commonly used in recent years. The more prominent SCZ gene expression signature in these individuals could reflect gene expression changes induced by APDs themselves, the illness itself, or a combination of both. An important consideration is that toxicological tests only reflect acute effects of APD use at the time of death rather than chronic APD intake, which can span several decades. APD-negative individuals are typically not naïve to APD treatment since practically all patients diagnosed with SCZ are prescribed APDs.

APDs exert complex effects on the brain transcriptome. Gene expression data from macaques treated with clozapine and haloperidol indicated that APD exposure could alter gene expression in the same direction as observed in human SCZ samples for some genes and the opposite direction for other genes. Although the macaque data reflect the transcriptional effects of APD intake alone, the data are limited by sample size, potential differences between species, and differences in the APD exposure (6 months for macaques versus typically many years for humans with variable regimens and compliance). Nonetheless, some convergent effects between APD exposure in macaques and SCZ in humans support the conclusion that APDs contribute to (and may thus confound) gene expression changes observed in SCZ. Interestingly, several modules with a convergent pattern of APD and SCZ effects (typically downregulated) were enriched for synaptic function. These modules also showed discrepancies between haloperidol and clozapine administration in macaques, with haloperidol effects being more convergent, and clozapine effects being less convergent or divergent, with SCZ effects. Although synapse-related genes are enriched in SCZ risk loci, our results suggest that the differential expression of these genes reported in postmortem brains from patients with SCZ may be related in part to the effects of APD exposure with some contrasting effects across different types of APD. Modules with a divergent pattern included those enriched for glucose homeostasis, mitochondria, and inflammatory response. These results are consistent with prior literature on dysregulation of energy metabolism in SCZ and complex actions of APDs on these pathways [20, 21], as well as potential modulation of neuroinflammatory response and activation states of microglia [22, 23]. While not all genes in these modules exhibited the same pattern, our findings support the notion that the therapeutic properties of APDs comprise metabolic and anti-inflammatory effects, not just the effects on synaptic transmission.

While APDs may contribute to differential gene expression in SCZ, the drugs do not appear to induce or reverse major cell-type shifts previously associated with SCZ. Our deconvolution of bulk gene expression data suggests that SCZ is associated with a reduction in GABAergic neurons and an increase in astrocytes. Interestingly, while cell-type enrichment of genetic risk loci [16, 24, 25] and recent single-nucleus RNA-seq case–control studies in SCZ [26–28] primarily implicate cortical pyramidal cells and GABAergic interneurons, our results are consistent with many previous marker-based and other studies of inferred changes in cell-type proportions [17, 29–32]. These inferred cell-type shifts occurred regardless of APD exposure. APD exposure alone also did not induce significant cell-type shifts in macaques. Cell-type deconvolution approaches can help infer major cell-type shifts but are affected by specific characteristics of the reference dataset and limited in their resolution of rare or closely related cell subtypes or cell-type-specific differential gene expression without significant shifts in overall cell number. For example, estimated proportions of microglia and oligodendrocyte precursors were not significantly changed, but some of their marker genes, *CX3CR1* and *PDGFRA*, were found in the “divergent” module M11 (Fig. 2b).

We have identified APD exposure as an important confounder in postmortem gene expression studies of SCZ patients. Several lines of evidence support the contribution of APDs to differential gene expression in SCZ. The widespread use of APDs in SCZ makes it difficult to account for APD exposure in human postmortem brain studies. Postmortem toxicology and medication history may help address this dilemma, but a more complete assessment of the transcriptional effects of APDs will require larger sample sizes of both human and non-human primates. Future studies should employ improved cell-type resolution (through use of single-nucleus profiling), assess other relevant brain regions [4] (such as basal ganglia and thalamus, which appear to be most affected by APD use [33, 34]), and integrate transcriptional data with genetic determinants of SCZ risk, APD response, and longitudinal changes in brain structure [35].

## Methods

### Human and macaque brain tissue samples

The human (CMC_HBCC) and macaque (CMC_macaque) brain specimens have been previously described [11]. Human brain samples were collected under protocols approved by the Institutional Review Boards (IRB) with permission of the next of kin. Clinical diagnoses were based on family interviews and a review of medical records using DSM-IV criteria. Unaffected controls were defined as having no history of a psychiatric condition or substance use disorder, and negative toxicology. The CMC_macaque data consisted of gene expression data from DLPFC of rhesus macaques treated with clozapine (5.2 mg/kg/day), low-dose haloperidol (0.14 mg/kg/day), high-dose haloperidol (4 mg/kg/day), or placebo for 6 months.

### Toxicology screening

Toxicology was performed on postmortem blood obtained at the time of autopsy as previously noted [36] via gas chromatography mass spectrometry (GC-MS) by the medical examiner and/or by National Medical Services (www.nmslabs.com). A forensic panel which includes several medications commonly used in psychiatry including antipsychotics, was run, but additional compounds were tested when information was available indicating that the patient was taking a medication that was not included in the panel. Toxicological screening for antipsychotics in blood and brain produce highly consistent results [37], although concentrations tend to be higher in brain compared to blood [38, 39]. Therefore, when a negative result for antipsychotics was detected in blood, the tests were repeated in cerebellar tissue. Two of the 25 samples that were APD-negative in blood but APD-positive in the cerebellum were removed from the analysis.

### Transcriptome data

RNA-seq counts from the CMC_HBCC and CMC_macaque studies were downloaded from the CommonMind Consortium portal on synapse.org and analyzed in R. For CMC_HBCC, we included gene expression data from DLPFC of cases with schizophrenia and unaffected controls with associated toxicology data; we excluded technical replicates and cases with age of death under 17 years (age of youngest SCZ case). Genes with counts-per-million (CPM) > 1 in ≥50% of the samples for CMC_HBCC and ≥3 samples in the CMC_macaque data were included. Trimmed mean of *M*-values (TMM) normalization was performed in edgeR [40] followed by further analysis using limma/voom [41, 42] and principal component analysis (PCA) on logCPMs. We removed two outlier samples with a PC score >5 standard deviations from the mean in two or more of the top 20 PCs in CMC_HBCC and one outlier sample with a PC score >3 standard deviations from the mean in two or more of the top 20 PCs in CMC_macaque. The demographics of both datasets are summarized in Tables 1 and 2.

**Table 1:** Overview of the human dataset.

Toxicology subgroups	*n*	Sex (f;m)	Ethnicity (AA;Cauc;Latinx;Asian)	Age (mean ± SD)	PMI (mean ± SD)	RIN (mean ± SD)	pH (mean ± SD)
Control.Neg.	113	28;85	62;113;3;2	42.6 ± 15.7	30 ± 14.2	7.6 ± 0.9	6.5 ± 0.3
SCZ.Neg.	23	7;16	13;23;0;0	49 ± 16	38.2 ± 17.2	7.4 ± 1.1	6.4 ± 0.2
SCZ.Pos.atypical	28	6;22	12;28;2;1	51 ± 13.2	29.7 ± 12.9	7.3 ± 0.7	6.4 ± 0.3
SCZ.Pos.mixed	10	6;4	6;10;0;0	45.6 ± 11.5	36.8 ± 17.5	7.6 ± 0.8	6.2 ± 0.1
SCZ.Pos.typical	27	11;16	20;27;0;1	52.4 ± 13.3	45.1 ± 33.3	7.3 ± 1	6.4 ± 0.2

*f* female, *m* male, *AA* African American, *Cauc* Caucasian, *SD* standard deviation, *Age a*ge of death in years, *PMI* post-mortem interval in hours, *RIN* RNA integrity number.

**Table 2:** Overview of the macaque dataset.

Group name	Treatment (mg/kg/day)	*n*	Sex (f;m)	Age (mean ± SD)	RIN (mean ± SD)
Placebo	Placebo (NA)	7	4;3	6.5 ± 1.3	7.6 ± 0.4
CLZ	Clozapine (5.2)	9	5;4	6.2 ± 1.5	7.4 ± 0.6
HAL.lo	Haloperidol (0.14)	10	5;5	6 ± 1.4	7 ± 0.7
HAL.hi	Haloperidol (4)	7	4;3	6.3 ± 1.2	7.3 ± 0.6

Acronyms as in Table 1.

### Differential gene expression

Weighted least-squares regression via limma/voom [41] was used to produce covariate-corrected residuals prior to differential gene expression. Covariates were selected based on a significant correlation with the top 20 PCs. Colinear covariates were regressed sequentially until no more significant correlations were found.

The model for CMC_HBCC was:

$$\begin{array}{ll}{\rm{Gene}}\;{\rm{expression}}\left( {{\rm{logCPM}}} \right)\sim {\rm{library}}\;{\rm{batch}} + {\rm{sex}} + {\rm{age}} + {\rm{effective}}\;{\rm{mapping}}\;{\rm{rate}} \\+\, {\rm{intergenic}}\;{\rm{rate}} + {\rm{RNA}}\;{\rm{integrity}}\;{\rm{number}}\left( {{\rm{RIN}}} \right) + {\rm{post}}\mbox{-}{\rm{mortem}}\;{\rm{interval}}\left( {{\rm{PMI}}} \right) + {\rm{pH}}\end{array}$$

The model for CMC_macaque was:

$$\begin{array}{ll}{\rm{Gene}}\;{\rm{expression}}\left( {{\rm{logCPM}}} \right)\sim {\rm{RNA}}\;{\rm{isolation}}\;{\rm{batch}} + {\rm{sex}} \\+\, {\rm{effective}}\;{\rm{mapping}}\;{\rm{rate}} + {\rm{intergenic}}\;{\rm{rate}} + {\rm{RIN}}\end{array}$$

The residuals from these models were then used to conduct differential gene expression tests in limma between SCZ cases and controls in CMC_HBCC or each treatment and placebo in CMC_macaque. *p* Values were adjusted for multiple testing via the Benjamini–Hochberg method.

### Aggregated score of differential gene expression (“SCZ expression signature”)

To aggregate differential gene expression in SCZ per sample across many genes, we projected (via dot product) covariate-corrected gene expression values for each sample onto the SCZ betas (i.e. log fold change) for all genes with a nominal *p* < 0.05 for SCZ.

$$\begin{array}{ll}{\rm{SCZ}}\;{\rm{expression}}\;{\rm{signature}}\\ =\, {\rm{gene}}\;{\rm{expression}}\;({\rm{logCPM}}\;{\rm{residuals}}) \cdot {\rm{SCZ}}\;{\rm{betas}}\left( {{\rm{log}}\;{\rm{fold}}\;{\rm{change}}} \right)\end{array}$$

A higher score indicates gene expression deviating from the mean in the direction of SCZ, while a lower score indicates changes in the direction of controls. To ensure that differences in the covariates such as age, sex, RIN, PMI, and pH were not driving the values, residuals from the above-mentioned regression models in limma were used for the score calculation.

### Validation dataset for human DLPFC

For validation of human DLPFC findings, we used a small case–control dataset (15 controls, 15 SCZ cases) with associated toxicology derived from brain tissue (cerebellum) as previously described [43]. Raw gene expression data was processed using adapter trimming via cutadapt and pseudoalignment to the human transcriptome (GENCODE v32) via Salmon [44]. Counts were summarized at the gene level, and only genes with CPM > 1 in ≥3 samples were included. One control sample was removed due to low RIN (<5). TMM normalization was performed in edgeR [40] followed by linear regression analysis via limma/voom [41]. The model used for covariate adjustment was:

$${\rm{Gene}}\;{\rm{expression}}\left( {{\rm{logCPM}}} \right)\sim {\rm{sex}} + {\rm{RIN}} + {\rm{pH}} + {\rm{PMI}}$$

Residuals from this model were used for differential gene expression tests between SCZ cases and controls in limma and for aggregating differential gene expression per sample by projecting them onto the SCZ betas for all genes with a nominal *p* < 0.05 for SCZ, as described above.

### Comparison between macaque and mouse APD effects

To compare the chronic effects of clozapine and haloperidol in macaque DLPFC to those previously described for the same APDs in another species, we used a mouse forebrain gene expression dataset consisting of animals treated with clozapine or haloperidol for 12 weeks or untreated controls (*n* = 10 per group) [12]. RMA-normalized Affymetrix mouse expression array MOE430A data were downloaded from GEO (accession: GSE6512) and processed using limma [42]. Normalized intensity values were averaged across multiple probes for the same gene and differential gene expression tests were performed comparing the clozapine and haloperidol groups against untreated controls in limma. For each APD group in macaques, the corresponding *t*-statistics for the genes with one-to-one orthologs between macaque and mouse are shown in Supplementary Fig. S5.

### Consensus WGCNA

To identify conserved functional gene co-expression networks shared between humans and macaques, we performed consensus WGCNA [14] between the two species. Gene expression data from CMC_HBCC and CMC_macaque were corrected for all technical covariates (i.e. library batch, effective mapping rate, intergenic rate, RIN, PMI, pH). Only genes with a one-to-one ortholog between the two species were used as input data. To obtain conserved co-expression modules with consistent directionality of effect, a consensus WGCNA was run with “signed” network type, soft thresholding power of 12, minimum module size of 30, deepSplit of 2, mergeCutHeight of 0.25, and minKMEtoStay of 0.3. The relationship of module eigengenes for each human and macaque sample were then investigated using a linear model:

$${\rm{Module}}\;{\rm{eigengenes}}\left( {{\rm{CMC}}\_{\rm{HBCC}}} \right)\sim {\rm{diagnosis}}$$

$${\rm{Module}}\;{\rm{eigengenes}}\left( {{\rm{CMC}}\_{\rm{macaque}}} \right)\sim {\rm{APD}}\;{\rm{treatment}}$$

### Functional enrichment

Gene ontology categories for each gene were obtained from BioMart (Ensembl version 86 [GRCh38] for CMC_HBCC and 75 [Mmul_1; GRCh37] for CMC_macaque) and tested for enrichment using Fisher’s exact test. All expressed genes served as the background distribution, further restricted to one-to-one matches for WGCNA module enrichment analyses. GWAS enrichment was tested using Fisher’s exact test based on overlap with prioritized genes (FINEMAP, SMR, rare damaging mutations) in the most recent SCZ GWAS [15].

### Cell-type deconvolution

Major cell-type proportions were estimated using Bisque [18] via the reference data-based method and all expressed genes as the input. For CMC_HCC, single-nucleus RNA-seq data from human DLPFC (manuscript under review; overlap of 5 samples was used to improve the model) served as a reference. For CMC_macaque, single-nucleus RNA-seq data from adult macaque DLPFC [19] was used as reference data (these data did not include microglia). Unsupervised clustering of single-nucleus data was visualized via uniform manifold approximation and projection (UMAP) plots using the top 20 principal components on the top 2000 most variable genes in Seurat (Supplementary Fig. S5a, b). In the macaque single-nucleus reference data, endocytes, and pericytes were combined, and clusters ExN9 and Astro2 were removed due to clustering separately from most excitatory neurons and astrocytes, respectively.

### Statistical analysis and plotting conventions

The distribution of aggregated SCZ differential gene expression scores and estimated cell-type proportions were tested for normality using a Shapiro–Wilk test. Both were significantly different from a normal distribution, and therefore non-parametric tests were used for all group comparisons (Mann–Whitney *U*-test with two groups; Kruskal–Wallis test with multiple subgroups; post hoc Dunn test for individual subgroup tests). Module eigengenes were tested for association with APD treatment in macaques and SCZ in humans using linear regression followed by two-sided *t* tests, although normality of module eigengene distribution was not formally tested. To account for multiple testing, we used the Benjamini–Hochberg false discovery rate (FDR) method. Differential gene expression results and effect sizes (log fold changes) are listed in Supplementary Tables S1 and S3. Fisher’s exact test results and odds ratios for gene ontology term over-representation are shown in Supplementary Table S3 for differentially expressed genes and Supplementary Table S4 for consensus WGCNA modules. All other statistical test results and effect sizes are listed in Supplementary Table S2. In all boxplots, the boxes extend up to the upper and lower quartiles, and whiskers extend up to 1.5 interquartile ranges from the upper and lower quartiles.

## Supplementary information

Supplementary Figures

Supplementary Table S1

Supplementary Table S2

Supplementary Table S3

Supplementary Table S4

## Data Availability

Data for CMC_HBCC and CMC_macaque are available at the CommonMind Consortium portal on synapse.org. Data for the validation dataset from UC Irvine were deposited to the Gene Expression Omnibus (GSE224683).

## Code availability

R code underlying the analyses performed here is available on GitHub (https://github.com/aschulmann/antipsychotics_signature).
