# Supplementary material for: Antipsychotic drug use complicates assessment of gene expression changes associated with schizophrenia
Source: Transl Psychiatry. 2023 Mar 17;13:93. doi: 10.1038/s41398-023-02392-8 (PMC10023659; doi:10.1038/s41398-023-02392-8)
Supplement: Supplementary file 1 — Supplementary Figures [file 41398_2023_2392_MOESM1_ESM.docx]

**Supplementary Figures**

**Supplementary Fig. S1 – SCZ expression signature for toxicological subgroups based on PsychENCODE SCZ betas.** SCZ expression signatures are shown for the same groups as in Fig. 1 but with signature calculated using SCZ betas (log fold changes) derived from PsychENCODE.

**Supplementary Fig. S2 – SCZ expression signature for toxicological subgroups in a UC Irvine validation dataset with associated brain toxicology.** SCZ expression signatures are shown for subgroups based on toxicological results: APD-negative controls (Control.neg; n=14), APD-negative SCZ cases (SCZ.neg; n=10), and APD-positive SCZ cases (SCZ.pos; n=5). APD-positive individuals shown in (a) are further subdivided by APD compound (b), namely clozapine (n=3) and quetiapine (n=2).


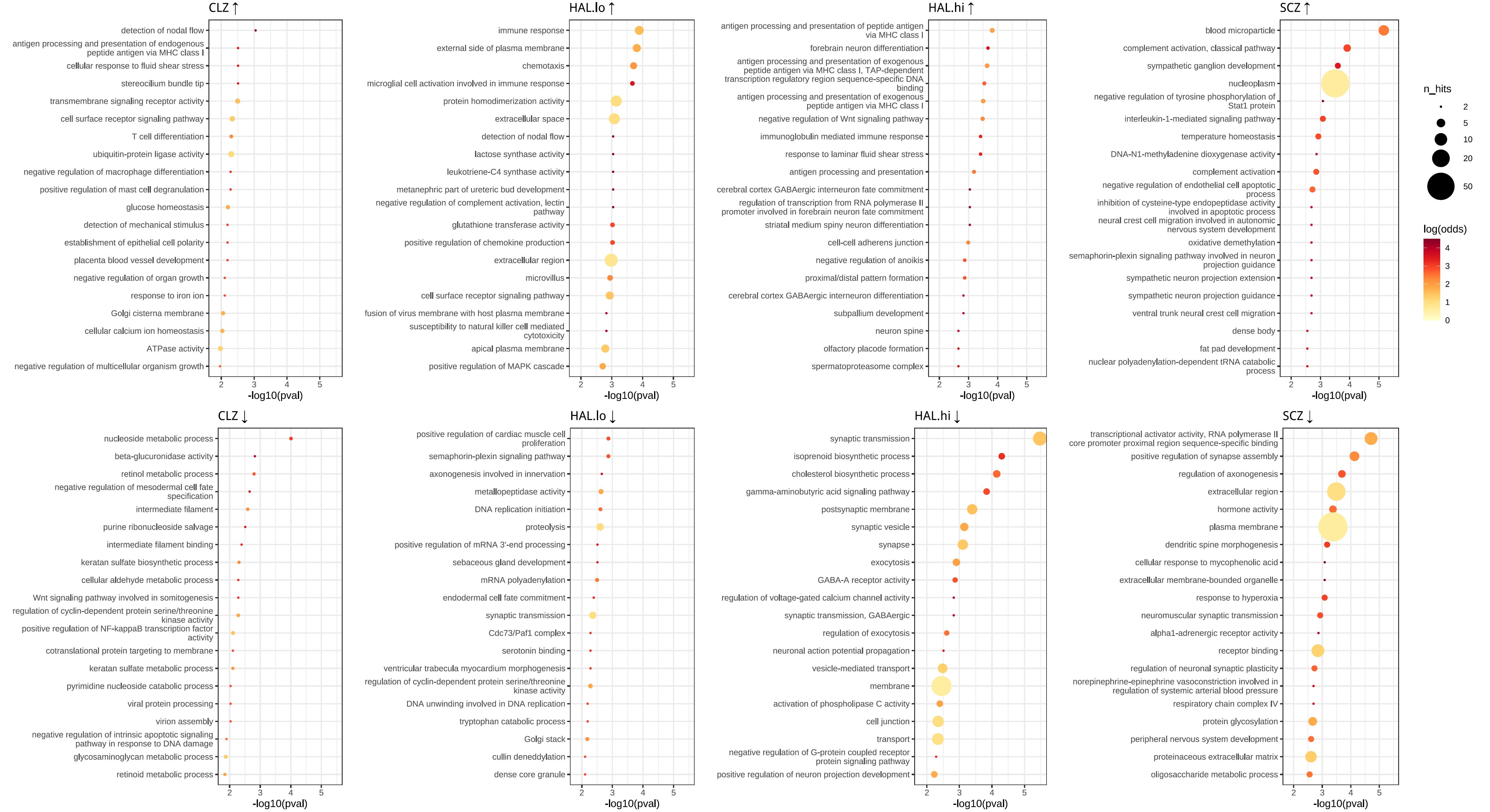


**Supplementary Fig. S3 – Gene ontology enrichment analysis for differentially expressed genes.** The top 20 overrepresented gene ontology terms are shown for genes associated with APD exposure in macaques and SCZ in humans. P-values are based on a two-sided Fisher’s exact test using the top 200 upregulated (top row) or downregulated (bottom row) genes, respectively. Dot size represents the number of overlapping genes and color indicates the strength of the enrichment represented as log(Odds Ratio). See also Supplementary Table S3.

**Supplementary Fig. S4 – Comparison between macaque and mouse APD effects.** Macaque differential gene expression t-statistics (y-axis) for each treatment group are plotted against the mouse differential gene expression t-statistic (x-axis) for the same APD. Macaque data are based on RNA-seq from DLPFC of animals treated with clozapine (a), low-dose haloperidol (b), and high-dose haloperidol (c) for 6 months. Mouse data are based on microarray data from mouse frontal cortex of animals treated with clozapine (a) or haloperidol (b,c) for 12 weeks. Linear regression lines with shaded 95% confidence intervals are shown along with Pearson’s correlation coefficients (R) are two-sided t-test p-values.

**Supplementary Fig. S5 – Additional analyses of estimated cell type proportions.** Unsupervised clustering of human (a) and macaque (b) single-nucleus RNA-seq data that were used as reference data is shown as a UMAP plot with annotated cluster name labels. Excitatory neuron and inhibitory neuron clusters were combined and macaque clusters ExN9 and Astro2 were removed prior to deconvolution due to clustering separately from other excitatory neurons and astrocytes, respectively. Inferred cell type proportions are shown in human bulk RNA-seq data for SCZ cases and controls (c) and in macaque bulk RNA-seq data for each treatment group (d). Results for excitatory neurons, inhibitory neurons, and astrocytes are shown in Fig. 3. For detailed statistical test results, see Supplementary Table S2.

**Supplementary Tables**

**Supplementary Table S1**: Differential gene expression for SCZ subgroups.

**Supplementary Table S2**: Statistical tests for SCZ expression signature, module eigengenes, GWAS enrichment, and cell type proportions.

**Supplementary Table S3**: Differential gene expression and gene ontology enrichment of top genes for APD treatment in monkeys and SCZ in humans.

**Supplementary Table S4**: Consensus WGCNA module membership and gene ontology term enrichment.
